# The Role of ABC Transporters in Lipid Metabolism and the Comorbid Course of Chronic Obstructive Pulmonary Disease and Atherosclerosis

**DOI:** 10.3390/ijms22136711

**Published:** 2021-06-23

**Authors:** Stanislav Kotlyarov, Anna Kotlyarova

**Affiliations:** 1 Department of Nursing, Ryazan State Medical University, 390026 Ryazan, Russia; 2 Department of Pharmacology and Pharmacy, Ryazan State Medical University, 390026 Ryazan, Russia; kaa-rz@ya.ru

**Keywords:** chronic obstructive pulmonary disease, COPD, atherosclerosis, inflammation, ABC transporters, lipid metabolism

## Abstract

Chronic obstructive pulmonary disease (COPD) ranks among the leading causes of morbidity and mortality worldwide. COPD rarely occurs in isolation and is often combined with various diseases. It is considered that systemic inflammation underlies the comorbid course of COPD. The data obtained in recent years have shown the importance of violations of the cross-links of lipid metabolism and the immune response, which are links in the pathogenesis of both COPD and atherosclerosis. The role of lipid metabolism disorders in the pathogenesis of the comorbid course of COPD and atherosclerosis and the participation of ATP-binding cassette (ABC) transporters in these processes is discussed in this article. It is known that about 20 representatives of a large family of ABC transporters provide lipid homeostasis of cells by moving lipids inside the cell and in its plasma membrane, as well as removing lipids from the cell. It was shown that some representatives of the ABC-transporter family are involved in various links of the pathogenesis of COPD and atherosclerosis, which can determine their comorbid course.

## 1. Introduction

Chronic obstructive pulmonary disease (COPD) is an important medical problem, which is due to the high prevalence of the disease, its impact on the quality of life, and the high incidence of disability and mortality [1,2,3]. An important characteristic of COPD is the variety of its clinical manifestations, which are based on a variety of not fully studied pathophysiological mechanisms [4,5,6]. This clinical heterogeneity has different pulmonary and extrapulmonary characteristics, such as the development of emphysema and other comorbidities [7,8]. Comorbid diseases make a significant contribution to the clinical picture of COPD [9,10,11]. It is considered that atherosclerosis and COPD have a number of common mechanisms of development, which allows us to consider them comorbid diseases [11,12].

COPD and atherosclerosis commonly occur simultaneously in the same patients, which mutually aggravates their course and prognosis [12]. The close relationship between COPD and atherosclerosis is well known to clinicians [13]. Both diseases occur relatively unnoticed for a long time and are often detected when they are already at clinically pronounced stages. In many cases, it is impossible to determine which of the diseases developed first. Diseases are also connected through restrictions on physical activity due to shortness of breath or pain, a decrease in the quality of life, an increase in the number of hospitalizations, and the risk of premature death. Diseases associated with atherosclerosis are a common cause of death in COPD patients [14]. The effect of the rate of bronchial obstruction increase and the increase in the number of COPD exacerbations on the progression of atherosclerosis is also well known [15,16,17]. In turn, the significance of lipid metabolism disorders on lung function in atherosclerosis is of great clinical and research interest. The lungs have a unique lipid biology that provides not only respiratory function but is also involved in the regulation of the immune response [18,19]. Disruption of the complexly maintained lipid homeostasis in the lungs is a part of COPD pathogenesis [20,21,22]. It is known that blood plasma lipids can be associated with pulmonary function [23,24,25]. For example, serum levels of oxidized low-density lipoprotein (ox-LDL) correlate with FEV_1_ (forced expiratory volume in 1 s) in patients with COPD, which is associated with its involvement in inflammation [26].

Interestingly, atherosclerosis is not often equally represented in different groups of patients with COPD but is more characteristic of the bronchitis phenotype, which suggests the presence of common disturbed mechanisms [27]. To date, there is no clear understanding of all the processes that connect the development and progression of COPD and atherosclerosis, but it is considered that the connecting link is systemic inflammation [27,28]. Many cells are involved in the maintenance of systemic inflammation, including macrophages, which are known for the diversity of their functions [28]. In both COPD and atherosclerosis, macrophages excessively infiltrate the bronchial or vascular wall, respectively [29,30,31].

Macrophages actively participate in the development of COPD [32,33,34,35,36]. They are also key participants in the initiation and progression of atherosclerosis, which is determined by their role in the uptake of modified lipoproteins in the arterial walls, and the production of inflammatory mediators and matrix metalloproteinases that contribute to the instability of atherosclerotic plaques [37]. A violation of the normal homeostasis of cholesterol in macrophages and a massive accumulation of its esters in lipid droplets lead to the acquisition of the so-called “foam cell” phenotype by cells [38]. Removing excess cholesterol from cells is called reverse cholesterol transport. This mechanism provides protection for the artery wall from unwanted lipid deposition, i.e., it has an antiatherogenic effect. Currently, the role of lipid metabolism disorder in the development of atherosclerosis is sufficiently understood and supported by the results of numerous studies.

Recent data have shown that lipid metabolism disorders are involved in many cross-immunometabolic pathways and participate in the development of COPD [39,40]. Furthermore, although the development and progression of COPD are based on the inflammation in the bronchi caused by long-term smoking, the results of studies indicate a deep metabolic rearrangement, both at the cellular level and in the patient’s body as a whole [39,41]. For example, the association of low body weight in COPD with high mortality is known, which has been called the “obesity paradox” [42,43,44]. Despite the fact that the prognostically significant cause of weight loss in this category of patients is the loss of muscle mass rather than fat, the importance of lipid metabolism disorders in COPD patients is not in doubt. As it is well known, excess body weight correlates well with an unfavorable prognosis in cardiovascular diseases of an atherosclerotic nature, but it has an inverse relationship with the prognosis of COPD [45], which is an important difference that characterizes the features of lipid metabolism in these patients.

**Figure 1:**
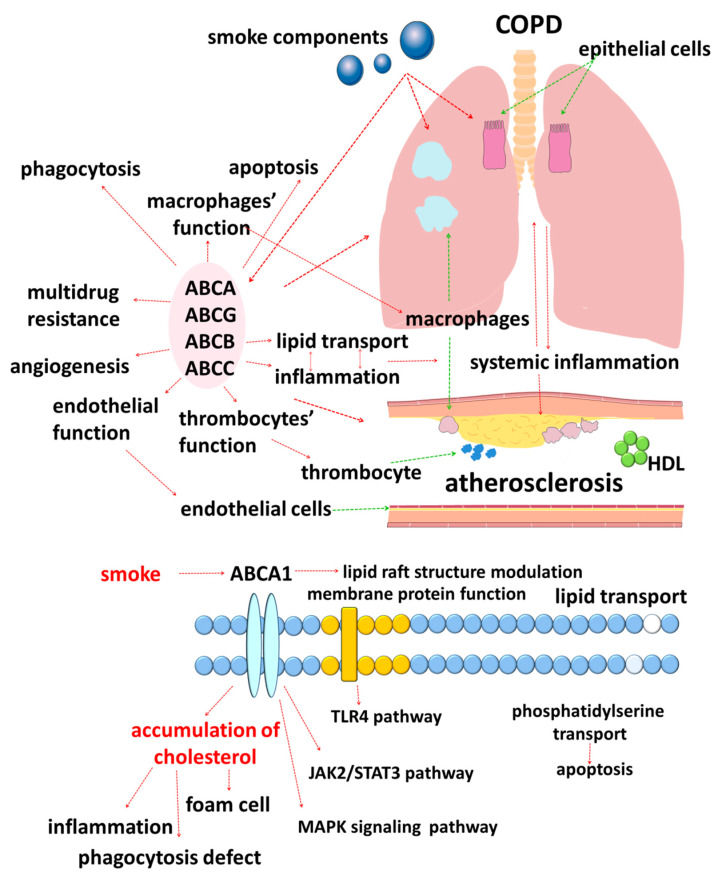
Schematic image of the involvement of ABC transporters in the pathogenesis of COPD and atherosclerosis.

The results of studies published in recent years suggest that cellular lipid metabolism has numerous cross-links with the immune response [46]. In this regard, disorders of lipid metabolism mediated by ATP-binding cassette (ABC) transporters are of clinical interest (Figure 1).

ABC transporters are a large family of membrane proteins that transport chemically diverse substrates through the lipid bilayer of cell plasma membranes while accompanied by ATP hydrolysis [47,48]. Currently, 49 different genes encoding ABC transporters are known in humans, which, based on the structural features, are divided into seven subfamilies, designated ABCA–ABCG [49,50,51,52,53].

The functions of many ABC transporters are not completely clear today. It is considered that about twenty of them are involved in the transport of lipids or lipid-like molecules, such as steroids (including cholesterol and bile acids), phospholipids, and sphingolipids [54,55,56]. Moreover, lipid carriers are present in all subfamilies of ABC transporters, which emphasizes the importance of lipid transport [54,55,56]. The need for special transport mechanisms for lipids is due to their insolubility in water [54]. In addition, cell membranes are heterogeneous in their lipid composition [55]. Thus, the outer leaflet of the membranes is rich in phosphatidylcholines and sphingolipids, while the inner leaflet is enriched with phosphatidylethanolamine and phosphatidylserine [54,55]. Unlike flippases, ABC transporters are responsible for the movement of lipid substrates from the inner leaflet of the plasma membrane to the outer leaflet, where lipids must be desorbed or diffuse to extracellular lipid acceptors [54].

It is interesting that lipids act not only as substrates for ABC transporters but can also regulate their transport activity by themselves. The accumulated data in recent years indicate an important role of cholesterol in the functioning of ABC transporters. Cholesterol is a key molecule of the plasma membrane that provides stabilization of the spatial structure of the lipid bilayer [57,58]. Cholesterol molecules are located in the plasma membrane in such a way that their hydroxyl groups are close to the glycerol region of the backbone of the lipid bilayer, while the hydrophobic rings are located in the hydrophobic core of the bilayer. Such a spatial arrangement of the molecules is considered to contribute to the participation of cholesterol in the regulation of the function of transmembrane proteins through two main mechanisms: direct interaction of the sterol with specific protein binding sites and indirect influence on the biophysical properties of the membrane [57,58,59,60]. This information is of great clinical and research interest since it allows us to assess the significance of separate processes in the development and progression of comorbid COPD and atherosclerosis in a different way.

The objective of this review was the analysis of the role of lipid metabolism disorders in the pathogenesis of the comorbid course of COPD and atherosclerosis and the participation of representatives of ABC transporters in these processes.

## 2. Subfamily of ABCA Transporters

The ABCA subfamily in humans includes 12 proteins that are well known for their participation in lipid transport and are subdivided into two subgroups by their structural and functional characteristics: ABCA1-like and ABCA6-like transporters [50,61].

The most well-known member of this subfamily to date, namely, ABCA1, was first described in 1994 and subsequently identified as the cause of Tangier disease. This rare genetic disease, which is found in only about 100 people in the world, is characterized by a violation of the formation of high-density lipoprotein (HDL) and the development of cardiovascular complications as a result [62]. Participation in the transport of cholesterol from the cell and the formation of HDL are considered key functions for the ABCA1 transporter. However, the molecular mechanisms of ABCA1-mediated lipid export and HDL formation, as well as data on all ABCA1 substrates, remain largely unclear. In this regard, several models of lipid export and subsequent HDL formation have been proposed [62,63,64,65].

ABCA1 is a full transporter consisting of four domains that are formed by a single polypeptide chain [50,66]. It was shown that although ABCA1 is localized in the plasma membrane of cells, it is able to recirculate between the plasma membrane and cell organelles, which ensures its cholesterol export function [67].

ABCA1 is expressed in various cells of many organs [68], including macrophages, where it is a key participant in the process of reverse cholesterol transport, ensuring its removal from cells. Recent data suggest an important role of reverse cholesterol transport in the function of macrophages.

These and other data gave the possibility to determine the important antiatherogenic function of the ABCA1 transporter in the formation of HDL by ensuring the processes of the reverse cholesterol transport from the cell, as ABCA1-dependent cholesterol efflux is the most important factor in the prevention of excessive accumulation of cholesterol in macrophages in the arterial wall and their transformation into “foam cells” [64,69].

**Figure 2:**
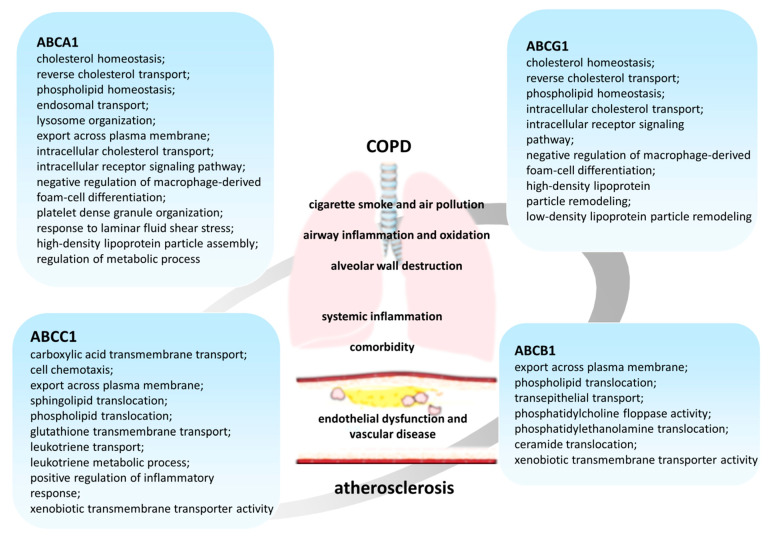
Biological processes and molecular functions of ABC transporters in the pathogenesis of COPD and atherosclerosis.

The data obtained in recent years allow us to consider that the function of the ABCA1 transporter is more multifaceted and is not limited to participation in the pathogenesis of atherosclerosis alone (Figure 2) [70]. The information about the participation of the protein in lung function is interesting, which is emphasized by the high levels of its expression in this organ. The data of the related studies have shown a decrease in the expression of ABCA1 in lung tissues in COPD [71,72,73]. This correlates well with the fact that smoking reduces the expression and functional activity of ABCA1. As a result, the reverse transport of cholesterol from the cell decreases, which leads to its intracellular accumulation (Figure 1).

Experiments on animals contributed to producing data on the role of ABCA1. In Abca1-knockout mice, pronounced changes in the lungs were detected, which included alveolar proteinosis, thickening of interalveolar septa, the formation of foamy alveolar macrophages, and hyperplasia of alveolar pneumocytes of type II [74].

These changes progressed with age and were characterized by the destruction of the alveolar architecture and epithelization of the remaining alveoli due to type II pneumocyte hyperplasia [75].

Thus, a decrease in the lipid transport function of ABCA1 in macrophages, both in the bronchi and in the vascular wall, is an important link in the development of COPD and atherosclerosis. Although no parallels have been established between these processes in the lungs and arteries, the current understanding of the function of ABCA1 in the pathogenesis of COPD and atherosclerosis is based on the fact that reverse cholesterol transport not only provides cellular and extracellular cholesterol homeostasis but also participates in inflammation through several mechanisms. These mechanisms include the regulation of the TLR4 (Toll-like receptor 4) signaling pathway, which provides an inflammatory response to the lipopolysaccharide (LPS) of the cell wall of Gram-negative bacteria and is widely represented in the cytoplasmic membrane of various cell types, including macrophages, and is actively involved in the pathogenesis of both COPD and atherosclerosis. It is considered that tobacco smoke promotes TLR4 activation. The participation of ABCA1 in the regulation of TLR4 is carried out through a change in the cholesterol content in the so-called lipid rafts, domains of the plasma membrane into which signaling molecules, such as TLR4, are recruited [76].

Excessive intracellular accumulation of cholesterol in macrophages due to a decrease in the transport function of ABCA1 contributes to the initialization of NLRP3 (NLR family pyrin domain containing 3) inflammation, which makes a significant contribution to the progression of atherosclerosis. It should be noted that although NLRP3 inflammation is one of the key links in the pathogenesis of COPD, unlike atherosclerosis, there is currently no evidence that it can be initiated by the accumulation of cholesterol in lung macrophages [77,78,79].

ABCA1 may also be involved in inflammation through the regulation of the JAK2/STAT3 (Janus kinase 2/signal transducer and activator of transcription 3) pathway, which is connected by the IL-6 (interleukin-6) signaling pathway [80,81], which is a cytokine that is well known in the pathogenesis of COPD and atherosclerosis. The JAK2/STAT3 pathway can perform both an anti-inflammatory function in macrophages [80,82] and demonstrate a proinflammatory effect [80,81,82,83,84]. It was shown that JAK2/STAT3 is involved in the regulation of airway inflammation in COPD [85].

Several recent studies have shown that ABCA1 also participates in the regulation of inflammation by inhibiting the secretion of interleukin-1β (IL-1β) [86] and tumor necrosis factor (TNF-α) [87].

Thus, smoking, which changes the transport activity of ABCA1, can participate in the violation of the lipid homeostasis of cells and contribute to the initialization and generalization of inflammation [88]. The assumptions about the participation of the transporter in the systemic mechanisms can be supported by observations in which a decrease in the level of mRNA ABCA1 in circulating white blood cells is determined in patients with atherosclerosis as compared to a control group [89].

The mentioned data may generally indicate the presence of a link between COPD and atherosclerosis, although they do not explain why the same etiological factor, namely, cigarette smoking, leads to the development of different COPD phenotypes.

Taking into account the significance of metabolic syndrome and overweight in atherosclerosis, it was shown that obesity and insulin resistance are associated with lower ABCA1 expression in visceral adipose tissue [90,91]. Based on these and other data, ABCA1 was proposed as a candidate gene for metabolic syndrome [92]. In addition, in skeletal muscle cells, ABCA1 may promote an increase in glucose uptake by enhancing Akt phosphorylation and transferring GLUT4 (glucose transporter type 4) to the plasma membrane [87].

A decrease in the functional activity of ABCA1 in skeletal muscles contributes to the abnormal accumulation of cholesterol and a decrease in glucose transport under conditions of insulin resistance [93]. The mentioned data may indicate the involvement of ABCA1 in the processes that determine the extrapulmonary clinical heterogeneity of COPD, taking into account the interesting relationships between the prognosis of the disease and body weight.

Other important mechanisms in which the transporter participates are the processes of apoptosis and phagocytosis, violations of which are well known in COPD [94,95,96]. Apoptosis also plays an important role in the pathogenesis of atherosclerosis: in early lesions, apoptosis is antiatherogenic, and in progressive lesions, apoptosis of macrophages contributes to atherogenesis [97,98,99]. It is considered that ABCA1 participates in the mechanisms of apoptosis through the presentation of the phosphatidylserine signaling molecule by apoptotic cells. Phosphotidylserine transferred to the outer side of the plasma membrane is considered one of the universal mechanisms for recognition, i.e., the so-called “eat-me signal” [100].

Another function of ABCA1 in phagocytosis is that it enhances the reverse transport of cholesterol in phagocytic active macrophages, providing a reduction in the cholesterol load of the macrophage after the absorption of apoptotic cells. It is known that ABCA1-deficient cells are less effective phagocytes [67,76], as a result of which, a decrease in the functional activity of the transporter can lead to the accumulation of apoptotic cells, which, in turn, can stimulate an inflammatory reaction [88].

The data on the participation of ABCA1 in platelet function and ensuring adequate hemostasis are interesting, considering the significance of hemostatic disorders in COPD and atherosclerosis. A decrease in the expression of ABCA1 leads to defects in platelet aggregation due to regulation impairment of vesicular granule transfer from the Golgi compartment to the plasma membrane [101,102,103]. Ultrastructural defects of platelets have also been described in patients with Tangier’s disease [102]. Interestingly, the absence of Abca7, a structurally close relative of Abca1, did not significantly affect the bleeding time from the tail of mice, which once again highlights the functional differences of the transporters [101].

Thus, the closely intertwined disorders of ABCA1-mediated cellular lipid efflux, homeostasis of membrane lipid rafts, and inflammatory activation of macrophages make a significant contribution to the pathogenesis of COPD and atherosclerosis.

It should be noted that, currently, there is insufficient information about the possible role of other members of the subfamily in the pathogenesis of COPD and atherosclerosis. At the same time, some of them are well known for their participation in lipid metabolism.

In recent years, there has been interest in ABCA7, another member of the subfamily, which is the closest homolog of ABCA1 and has some similarities in its functions since it carries out lipid transport and participates in the formation of HDL [104,105,106,107,108,109]. ABCA7 is expressed in macrophages [105,106,110,111], where it is localized both in the plasma membrane and intracellularly [108,112,113,114,115]. Several studies have shown the possible participation of ABCA7 in phagocytosis [111,116,117]. This understanding is based on the analysis of the CED-7 protein role in the nematode *Caenorhabditis elegans*, which functions in both phagocytic and apoptotic cells during phagocytosis and is necessary for the clustering of CED-1, which is a transmembrane receptor that initiates uptake signals. It was shown that CED-7 participates in the exposure of phospholipid ligands on the surface of apoptotic cells [118]. It was shown that ABCA7 has a high similarity of sequences to CED-7 and is required for effective phagocytosis of apoptotic cells [110,116]. However, experiments on Abca7 knockout mice did not show any visible lung abnormalities. The females of these mice were characterized by a lower visceral fat mass and had lower levels of total serum cholesterol and HDL cholesterol [107]. Moreover, these disorders were less pronounced than in mice lacking Abca1, which indicates differences in the substrate specificity of the transporters [107].

Another member of the subfamily, namely, ABCA3, is well known for its participation in the formation of a surfactant [119,120], which is located in the alveoli at the air-tissue interface and ensures the normal function of the alveoli, preventing their collapse [121,122,123]. The transporter is expressed by type II alveolar pneumocytes and is localized in intracellular lamellar bodies [61,124,125]. Known substrates for the ABCA3 transporter are phosphatidylcholine, phosphatidylglycerol, and cholesterol [126,127]. Although there is currently no data on the participation of ABCA3 in the pathogenesis of COPD [128], it is known that mutations in the ABCA3 gene can lead to lung fibrosis and the development of emphysema [120,129]. Most mice with Abca3 deletion in respiratory epithelial cells die shortly after birth from respiratory distress caused by surfactant deficiency, and emphysema was being developed in the absence of signs of severe pulmonary inflammation in the surviving mice [119,122,127,130,131,132]. Interestingly, STAT3, which is activated by IL6, regulates the expression of ABCA3 and affects the formation of lamellar bodies in alveolar type II pneumocytes [109,133].

The results of a recent bioinformatic analysis showed a decrease in the expression levels of ABCA13 in the bronchial epithelium in smokers [134]. These data may be of interest since, in humans, the transporter has the highest amino acid sequence homology with ABCA1 (50%), ABCA4 (50%), and ABCA12 (56%) [135]. In accordance with this, it is assumed that a violation of the expression of ABCA13 in smokers may lead to a violation of lipid transport processes and, related with them, functions of the innate immune response [134]. It should be noted that ABCA13 is known for its association with cancer, and the presented data may reflect the negative carcinogenic effect of smoking [134,136,137,138].

At the moment, the biological role of representatives of the group of ABCA6-like transporters, which include ABCA5, 10, 6, 9, and 8, is not understood well enough [109,139,140]. The potential participation of ABCA6, 9, and 10 in the processes of lipid transport in macrophages is assumed [61,141,142]. ABCA6 and ABCA5 are expressed in the lungs; moreover, ABCA5 is localized in lysosomes and late endosomes of type II alveolar pneumocytes, along with ABCA3 [140,143,144,145]. At the same time, abnormalities in the lungs are not revealed in *Abca5^−/−^* mice, but changes similar to cardiomyopathy develop in the heart [140,146]. In addition, the ABCA5-transporter is found in monocytes and macrophages, where it participates in cellular lipid metabolism, but the role of disorders of its function is still unclear. Interestingly, in vascular endothelial cells, mRNA ABCA6 transcription was stimulated by cholesterol and inhibited by insulin-like growth factor 1. These data allow us to suggest the potential participation of ABCA6 in intracellular lipid transport processes in endothelial cells [125].

Thus, representatives of the ABCA subfamily make a great contribution to the regulation of lipid transport processes. ABCA1 plays a particularly significant role in these processes, participating in the pathogenesis of COPD and atherosclerosis, which most studies are devoted to. However, it should be noted that for many representatives of the subfamily, the biological function is not fully known and requires further research.

## 3. Subfamily of ABCB Transporters

Members of the ABCB subfamily are well known to clinicians and researchers for their role in the development of multiple drug resistance. At the same time, a number of members of the ABCB subfamily are also characterized by their ability to transport lipids, such as ABCB1 [55,56,147,148], or ABCB4, which participates in the translocation of phosphatidylcholine lipids into bile [56,149,150].

ABCB1 (P-glycoprotein, MDR1 (multidrug resistance 1 gene)), cloned by J.R. Riordan in 1985, was originally described as a major participant in the mechanism of multiple drug resistance to chemotherapeutic agents of colchicine-resistant Chinese hamster ovarian cells [151,152,153]. To date, ABCB1 is one of the most well-studied ABC transporters (Figure 2) [55,56,154,155]. Its broad substrate specificity allows it to carry out the transport of chemically diverse molecules, including medicinal substances [56,147,156]. However, the mechanism by which ABCB1 can recognize different chemical structure substrates remains largely unknown.

It is known that ABCB1 participates in lipid transport [55,56,157], moving lipids from the inner to the outer leaflet of the plasma membrane of cells [56,76]. It was shown that cholesterol is recognized and transported as an endogenous ABCB1 substrate [158,159,160]. Moreover, ABCB1 not only transports cholesterol across the membrane but also its functional activity is modulated by cholesterol in the membrane [159,161]. A direct interaction between lipid molecules and ABCB1 was revealed [56,76,162].

It was shown that the predominant localization of ABCB1 in lipid rafts is necessary for its functioning [160,163,164]. Cholesterol depletion, which leads to the destruction of lipid rafts, disrupts the membrane localization of ABCB1 and reduces its transport activity [165,166], which leads to the intracellular accumulation of drugs in cells [159]. Cholesterol can bind directly to the active center of the transporter or influence it allosterically to adjust its size to the substrate. There is a supposition that sterols may interact with ABCB1 and modulate its structure and function by occupying part of the drug-binding pocket or by binding to assumed consensus cholesterol-binding motifs (CRAC/CARC) that are located in the transmembrane domains [55,58,60,167,168]. Thus, the effect of cholesterol on the transport activity of ABCB1 is of great clinical interest.

Although the lungs are not among the organs with a high expression of ABCB1, the protein is found in the bronchi, where it is mainly localized on the apical surface of the ciliated epithelial cells, the apical and lateral surfaces of the serous cells of the bronchial glands, and the lumen surface of the endothelial cells of the bronchial capillaries, but it is not found in the mucus-secreting goblet cells [169,170]. In the alveoli, ABCB1 is expressed in type I alveolar epithelial cells [171,172].

ABCB1 is activated during differentiation from monocytes to macrophages and is sensitive to activation by LXR (liver X receptor) agonists [173,174]. In addition, it is also present in macrophages, with higher expression in the M2 subtype compared to M1 macrophages [163,175,176,177]. It has been reported that tobacco smoke components affect the expression and functional activity of ABCB1 [172,178]. A decrease in the expression of *ABCB1* in the cells of smokers compared to non-smokers was shown. Moreover, a tendency toward a negative correlation of *ABCB1* expression with an increase in the number of packs-years and the number of cigarettes smoked per day was observed [170].

It should be noted that the inhaled administration of glucocorticoids in patients with severe COPD may cause existing differences in the expression of *ABCB1* in the lungs [179].

It has been shown that polymorphism of the *ABCB1* gene can affect the effectiveness of COPD therapy [180] and mediate extrapulmonary complications of the disease [181]. In addition, elevated levels of mRNA ABCB1 were found in the tissues in atherosclerosis, which allows us to suggest a role of ABCB1 in the development of atherosclerotic lesions in vivo [174,182].

Another member of the subfamily, namely, ABCB4, participates in phosphatidylcholine transport and the formation of bile in the liver [183]. The expression of mRNA ABCB4 in monocytes and macrophages is also shown [184,185], and the ABCB4 of macrophages prevents the formation of “foam cells,” reducing the accumulation of lipids and providing an atheroprotective function [185]. A lower serum HDL cholesterol level was observed in Abcb4 knockout mice fed with normal food, which confirmed the effect of ABCB4 on cholesterol metabolism [185,186]. Since ABCB4 is a carrier of phosphatidylcholine from the inner to the outer sheet of the plasma membrane, violations of the functional activity of the protein can probably lead to asymmetry of the phospholipid membrane of macrophages [185,187]. Thus, ABCB4 can indirectly contribute to atherogenesis by affecting the accumulation of lipids in macrophages as a result of modulation of the lipid membrane asymmetry [185].

The results of bioinformatics analysis indicate that exposure to cigarette smoke is associated with increased levels of *ABCB6* expression in bronchial epithelial cells, while smoking cessation is associated with lower *ABCB6* expression [134].

Literature data suggest that ABCB6 protects cells from oxidative stress by modulating cytosolic reactive oxygen species [188,189]. Despite this, an increase in the expression of *ABCB6* may also have negative effects on the form of increased resistance to chemotherapeutic agents [134].

ABCB6 may also be involved in atherogenesis [190,191]. It is known that platelets participate in the pathogenesis of atherosclerosis [192,193]. Activated platelets deposit cytokines, such as CCL5 (chemokine (C-C motif) ligand 5) or CXCL4 (chemokine (C-X-C motif) ligand 4), on the surface of endothelial cells, facilitating the recruitment of leukocytes to inflammatory foci, and also form aggregates with neutrophils and monocytes, which plays a key role in the production of inflammatory cytokines, the biosynthesis of leukotrienes, and the production of reactive oxygen species [193,194,195,196]. Platelets originate from megakaryocytes, where the latter comes from megakaryocyte progenitor cells in the bone marrow and spleen [193,197]. A high level of ABCB6 is observed in megakaryocyte progenitor cells and its deficiency leads to an increase in the number of circulating platelets, the interaction of platelets with inflammatory leucocytes, and the accelerated development of atherosclerosis [198].

Thus, representatives of the ABCB family are of great clinical importance, not only due to their involvement in the efflux activity for xenobiotics but also due to the movement of endogenous substrates.

## 4. Subfamily of ABCC Transporters

ABCC1 (MRP1 (multidrug resistance-associated protein 1)) was initially identified as a glutathione-conjugate transporter [55,199,200], but later its participation in lipid transport [55,201] and inflammatory responses was described (Figure 2).

In the lungs, ABCC1 is found in alveolar macrophages, as well as in bronchial epithelial cells [202], and its expression levels differ in different parts of the respiratory tract [134]. Localization of the protein in the cells of the distal parts of bronchi was already associated with participation in the development of COPD [203]. Interestingly, in contrast to ABCB1, ABCC1 in basal cells was distributed along the entire circumference of the plasma membrane, and in ciliated cells, it is localized on the basolateral surface [204], which predetermines the functional differences of the transporters [205].

ABCC1 is localized in cholesterol-rich actin-dependent lipid rafts, which determines the functional dependence of the efflux activity of the transporter on cortical actin [206,207]. Cortical actin stabilizes not only the lipid rafts but also the ABCC1 located in them. However, there is little information in the literature about the participation of cholesterol in the modulation of the ABCC1 transport function. In one study, it was found that the functional activity of the transporter decreased with a decrease in the level of cellular cholesterol [208], but in other studies, it was shown that cholesterol was not a necessary factor for the function of ABCC1 and, apparently, did not participate in the mechanisms of functional connection of ABCC1 with lipid rafts [209].

It was shown that ABCC1 can protect the lungs from developing COPD by reducing the oxidative stress caused by smoking, preventing the accumulation of toxic metabolites [210,211]. It is assumed that the expression of the *ABCC1* gene is regulated by a feedback mechanism since it is associated with oxidative stress and exposure to toxins caused by exposure to cigarette smoke and contributes to the enhancement of antioxidant activity in the epithelial cells of the respiratory tract [134].

ABCC1 may play an important role in smoking-related loss of lung function [212]. At the same time, information about the expression of *ABCC1* in COPD is ambiguous [179,213,214,215,216,217]. A lower *ABCC1* expression in the bronchial epithelium of COPD patients compared to healthy former smokers was shown [218]. Expression was also lower in patients with severe COPD than in those with mild or moderate COPD. An in vitro study also demonstrated that cigarette smoke extract inhibited ABCC1 activity in bronchial epithelial cells [219,220]. In contrast, data from a recent bioinformatics analysis show an increase in the expression of the *ABCC1* gene in smoking patients with COPD compared to people who do not have COPD [134]. The available contradictory information on the expression of the transporter in patients with COPD may be associated with the use of glucocorticoids in the severe course of disease [214]. However, obviously, these and other data indicate the involvement of the transporter in the development of COPD.

The association of ABCC1 with inflammation is interesting. Leukotrienes, which are a group of highly effective lipid mediators, are important participants in the antibacterial protection of the lungs. Their synthesis is induced by different microorganisms [221,222]. Leukotriene LTC_4_ occupies an important place among the physiological substrates of ABCC1 [200,223,224]. In this connection, the transport activity of ABCC1 may participate in pulmonary inflammation. It was shown that Abcc1^−/−^ mice are more resistant to Streptococcus-pneumoniae-induced pneumonia than wild-type animals [225]. They showed reduced pneumococcal growth in the lungs and strongly reduced mortality, which was associated with an increase in circulating LTB_4_, which is a powerful chemoattractant for neutrophils and increases the activity of phagocytic cells [226,227,228]. The main source of LTB_4_ in the lungs is alveolar macrophages [229,230]. It was shown that due to the absence of Abcc1, elevated intracellular levels of LTC_4_ increase the generation of LTB_4_.

In addition to these data, it was shown that mice with triple knockout of the *Mrp1* and *Mdr1a/1b* genes were more susceptible to the development of COPD. These mice had lower levels of IL-8 production and showed an almost complete absence of inflammatory cells in response to cigarette smoke [215,223]. The impaired inflammatory response was likely associated with lower LTC_4_ excretion [231].

Data about the participation of ABCC1 in the export of sphingosine-1-phosphate (S1P), which is a lipid mediator that is involved in many processes, including inflammation, angiogenesis, apoptosis, and macrophage function, are of great importance [232,233,234,235,236,237,238,239,240,241]. S1P also regulates the integrity of the endothelial barrier by modulating the endothelial cytoskeleton [242].

It was shown that S1P levels were elevated in the induced sputum of COPD patients compared to non-smokers [243]. It was suggested that S1P may be a participant in defective phagocytosis by macrophages in COPD [243,244].

Interestingly, the impairment of S1P metabolism is an important factor determining the emphysematous phenotype in COPD [245].

In addition to ABCC1, ABCA1 and ABCG2 also participate in S1P transport [234,246,247].

The participation of ABCC1 in the pathogenesis of atherosclerosis is currently the subject of active research. It has been determined that ABCC1 plays a definite role in the regulation of vascular endothelial homeostasis and arterial blood pressure by inducing the release of glutathione from vascular endothelial cells [248,249,250]. There are also reports on the direct involvement of the transporter in the process of atherogenesis [248,250]. ABCC1 may contribute to the occurrence and progression of cardiovascular diseases, and the inhibition of ABCC1 may represent a new strategy for the prevention of hypertension, endothelial dysfunction, and atherosclerotic vascular disease in high-risk patients with cardiovascular diseases [191]. ABCC1 is found in large quantities in vascular smooth muscle cells, which make up the majority of vascular wall cells and are involved in the process of atherosclerosis. ABCC1 acts as a transporter for substances such as glutathione, oxidized glutathione, and leukotriene C_4_ (LTC_4_) [248,250,251], which are potentially essential for regulating the production of reactive oxygen species in vascular cells. It was suggested that in the early stages of the development of an atherosclerotic lesion, the release of LTC_4_ under the action of ABCC1 from vascular smooth muscle cells may be of great importance. In the later stages, the main source of LTC_4_ is most likely macrophages. In addition, modulation of ABCC1 expression in human aortic endothelial cells affects vascular function [248,251].

Thus, ABCC1 demonstrates involvement in the development of COPD and atherosclerosis. However, the mechanisms of this participation require further study.

## 5. Subfamily of ABCG Transporters

It is known that many proteins of the ABCG subfamily also participate in lipid homeostasis [252]. An important role in lipid metabolism is assigned to ABCG1 and ABCG4 transporters, which are half-type ABC proteins since they consist of only one transmembrane domain and one nucleotide-binding domain. For activation to occur, the protein must form a dimer (homodimer or heterodimer) or even an oligomer depending on the function [253,254,255,256,257].

The most well-studied representative of this subfamily is ABCG1 (Figure 2). It is expressed in many cell types, including myeloid cells, lymphocytes, epithelial, and endothelial cells of various organs [258,259,260,261]. ABCG1 transports cholesterol, 7-ketocholesterol, sphingomyelin, and phosphatidylcholine from cells to HDL [255,258,262]. It is known that, like ABCA1, ABCG1 removes excess cholesterol from peripheral cells, saturating HDL with it and protecting cells from sterol overload [257,263,264]. According to the accepted model, ABCA1 provides primary saturation with cholesterol of ApoA-I, which is poor in lipids, thus forming “nascent” HDL, which is further lipidized by ABCG1-dependent cholesterol efflux [97,265,266,267,268]. Confirmation is that Abcg1^−/−^ macrophages express higher levels of Abca1 compared to wild-type macrophages in the tumor [269]. The results of recent studies have shown the significance of ABCG1 in the pathogenesis of COPD. In the lungs, *ABCG1* is expressed in various cell types, including alveolar macrophages, epithelial cells, and type II pneumocytes [258,270,271]. The absence of ABCG1 leads to progressive chronic lung inflammation, which is associated with impaired regulation of intracellular cholesterol levels [121,271,272]. Thus, massive infiltrates of lymphocytes, macrophages, and cholesterol crystals, as well as increased expression of many cytokines and cytokine receptors, appear in the lungs of an *Abcg^−/−^* mouse by 6–8 months after birth. In addition, there are signs of hemorrhage in the tissue. It was shown that inflammation in the lungs of Abcg1^−/−^ mice is a secondary process that develops in response to the accumulation of lipids [272]. In addition, the ratio of bacteria that inhabit the lungs is disrupted in mice lacking *Abcg1* [273].

It was shown that Abcg1^−/−^ macrophages were characterized by increased production of proinflammatory cytokines IL-6, IL-1β, IL-1α, and IL-12, and a decrease in anti-inflammatory cytokine IL-10 [272,274], which is associated with the lipid load of macrophages. Elevated levels of matrix metalloproteinases MMP-8 and MMP-12 were also found out in the lungs of *Abcg1^−/−^* mice [272], they destroy the extracellular matrix, are overexpressed in COPD patients, and are associated with airway inflammation and remodeling [275,276].

The information about the participation of ABCG1 in the polarization of macrophages is important. Experimental data indicate that ABCG1 deficiency contributes to the proinflammatory M1 polarization of human macrophages, and the molecular mechanism is probably mediated via the Akt signaling pathway [277]. Moreover, this phenotypic shift is more pronounced when the diet of mice was similar to the Western one [269]. According to the existing concepts, most of the cells in the center of the atherosclerotic plaque are M1 macrophages.

It is believed that ABCG1 participates in the apoptosis of cells, including macrophages [97,257,278,279]. It was determined that macrophages of mice *Abcg1^−/−^* have an increased ability to absorb apoptotic cells, accumulate lipids, and become apoptotic [274,280,281].

It is known that apoptosis plays an important role not only in the development of COPD but also in the pathogenesis of atherosclerosis: it is antiatherogenic in the early foci of lesions, and the apoptosis of macrophages contributes to atherogenesis in progressive lesions [97]. The mechanism of ABCG1 participation in apoptosis is probably determined by its cholesterol transport activity and its functioning as an inducer or inhibitor of apoptosis depends on the localization of the transporter on the plasma membrane or intracellular membranes of organelles [97].

Taking into account the presence of the ABCG1-transporter in macrophages (“foam cells”) in human atherosclerotic plaque, it was assumed that macrophagic ABCG1 plays an important role in the development of atherosclerotic lesions [174,282]. This assumption was confirmed in numerous studies [174,184,277,282,283,284]. The participation of ABCG1 in lipid homeostasis was analyzed by a number of authors who demonstrated that mRNA expression and ABCG1 synthesis in macrophages can be induced by cholesterol loading [97,184,284,285]. The data showing that ABCG1 is present in endosomes and participates in their cholesterol homeostasis are also interesting. Thus, ABCG1 is also important for intracellular cholesterol transport [286,287].

Another member of the subfamily, ABCG4 has 69% identity and 84% similarity in amino acid composition to ABCG1 and mediates the outflow of cholesterol to HDL-like ABCG1 [257,260,262,265]. However, unlike ABCG1, the expression of ABCG4 is limited. The transporter is found out in the brain and hematopoietic organs [260,288,289,290,291]. Another important difference is that the activation of LXR induces the expression of Abcg1, but does not affect the expression of Abcg4 [290].

The obtained results allowed for suggesting the role of ABCG1 and ABCG4 transporters in cell proliferation, apoptosis, and immune responses, and that these different processes may be related to the regulation of lipid metabolism [262,292,293].

Currently, there is no information about the possible participation of ABCG4 in the development of COPD. Although ABCG4 is not expressed in macrophage foam cells, its potential role in atherogenesis is described [294]. ABCG4 is present in bone marrow megakaryocyte progenitors and, like ABCG1, protects cells from sterol overload. It is known that ABCG4 inhibits the proliferation of megakaryocyte progenitor cells via reducing the transmission of thrombopoietin receptor signals in lipid rafts [257,295]. In addition to increased atherogenesis, arterial thrombosis was found in mice with *Abcg4* gene knockout, which correlated with an increase in the number of reticular platelets, platelet complexes, leucocytes, and microparticles of platelet origin, which have proven pro-atherosclerotic and prothrombotic properties. Researchers associated increased platelet production caused by impaired cholesterol metabolism in progenitor cells with accelerated atherogenesis and arterial thrombosis [67,295].

ABCG2 (BCRP, breast cancer resistance protein) is another representative of the ABCG subfamily and is most commonly associated with drug excretion [55,296]. BCRP has been found in many organs and tissues, including the endothelium of venous vessels and capillaries, where it performs a protective and barrier function [297].

The expression of ABCG2 in the lungs is low and decreases in the trachea–large bronchi–small bronchi series, but the protein is found in the epithelial layer and seromucinous glands, as well as in the endothelial cells of capillaries [298]. ABCG2 is also expressed in alveolar pneumocytes and is mainly localized on the apical membrane and, to a lesser extent, in the cytosol and in the cell nuclei, where, according to some assumptions, it acts as a transcription factor, regulating gene expression. ABCG2 is expressed differently in type I and type II alveolar pneumocytes, with greater severity and activity in the latter. During differentiation from alveolar pneumocytes of type II to alveolar pneumocytes of type-I-like phenotype, the expression of mRNA ABCG2 decreases [298,299,300,301].

The role of BCRP/ABCG2 in the lungs is completely unknown. Furthermore, lung pathologies in Abcg2^−/−^ mice have not been reported yet [176,302,303,304].

It has been shown that ABCG2 in lung tissues is responsible for the formation of the SP-phenotype (side population) of lung cancer cells, which have high efflux activity [305].

It was found in numerous studies that BCRP also participates in the transport of sterols, and cholesterol can stimulate the ATPase activity of the transporter [55,294,306,307]. This was confirmed by the fact that a change in the structure of lipid rafts directly leads to the redistribution of the BCRP protein in areas with a higher cholesterol density. With a decrease in the cholesterol content in the lipid rafts, the content of BCRP substrates in the cells increases, which indicates the inhibition of the transporter protein. The restoration of cholesterol and its saturation of lipid rafts leads to the normalization of the functional activity of the transporter [308,309].

ABCG5 and ABCG8 play a role in lipid metabolism and mediate the outflow of cholesterol and sitosterol from the intestinal walls and hepatocytes to the bile duct and intestinal lumen [159,310]. There is no information in the literature about the participation of these transporters in lung function. However, it is known that mutations of the *ABCG5* and *ABCG8* genes cause sitosterolemia, which is characterized by an increase in the absorption of plant and fish sterols, and their reduced biliary excretion leads to an increase in the level of toxic sitosterols in the blood and the early development of atherosclerosis and myocardial infarction. The participation of ABCG5 and ABCG8 in atherogenesis may also consist in the fact that they provide trans-intestinal excretion of cholesterol, i.e., an alternative non-biliary route of its excretion [311].

Thus, representatives of the subfamily are actively involved in lipid transport and perform many important functions. In the pathogenesis of COPD and atherosclerosis, ABCG1 is one of the most interesting since its transport activity disturbances disrupt a number of processes associated with the development of inflammation.

## 6. Conclusions

Recent studies confirm that COPD and atherosclerosis do not just occur simultaneously; both diseases have a great mutual influence, largely determining the nature of progression. Interestingly, the prevalence and severity of atherosclerosis are not equally represented among patients with different COPD phenotypes. In addition, depending on the body mass index, the prognosis of COPD has features that distinguish it from cardiovascular diseases. The well-known association of weight loss with the development of emphysema is also described. These findings open up a new perspective on the metabolic-mediated mechanisms participating in COPD development.

The performed analysis showed a significant role of lipid metabolism disorders in the pathogenesis of the comorbid course of COPD and atherosclerosis. It was shown that a number of members of the ABC-transporter family perform important functions in maintaining lipid homeostasis [312]. Their participation in the pathogenesis of COPD and atherosclerosis is multifaceted, as transporters are involved in many processes and functions of various cells involved in the processes of inflammation (Table 1).

The information of interest is that cholesterol is able to regulate the transport activity of some ABC transporters. Change in the concentration of cholesterol and its distribution in cell membranes may represent a mechanism for modulating the function of transmembrane proteins.

To date, the function of ABCA1 and ABCG1 transporters is best known in the pathogenesis of the comorbid course of COPD and atherosclerosis. Changes in their transport activity disrupt a number of processes associated with inflammation. Inflammation can develop via several mechanisms and can be caused by the accumulation of cholesterol in macrophages.

It should be recognized that for many ABC transporters, the biological role is not sufficiently clear and requires study. Obviously, we are still at the beginning of studying all the functions of lipid metabolism and the significance of their disorders.

Thus, this review, which aimed at taking a fresh look at the possible functions of ABC transporters in the development of COPD, showed that lipid metabolism mediated by ABC transporters and its disorders can make a large contribution to the nature of the progression and comorbid course of COPD.

## Figures and Tables

**Table 1:** Participation of key representatives of ABC transporters in lipid metabolism and the pathogenesis of COPD.

Transporter	Lung Cells Type	Lipid Substrates	Functional Role in the Pathogenesis of COPD	Link to COPD and Atherosclerosis	References
ABCA1	Macrophages;	Cholesterol;	Inflammation;		[62,63,64,65,67,68,69,70,71,72,73,74,75,76,80,81,84,86,87,88,89,93,101,102,103]
	alveolar type I pneumocytes;	phosphatidylcholine;	phagocytosis;		
	alveolar type II pneumocytes;	sphingosine-1-phosphate;	apoptosis;	High	
	bronchial epithelium;	sphingomyelin	macrophages		
	endothelial cells;		function;		
	granulocytes;		lipid rafts		
	airway smooth muscle cells		regulation;		
			trans-membrane		
			protein activity		
ABCB1	Endothelial cells;	Cholesterol;	Multidrug	Low	[55,56,157,158,159,160,161,162,163,170,172,173,174,175,176,177,178,179,180,181,182]
	alveolar type I pneumocytes;	phosphatidylcholine;	resistance;		
	macrophages;	ceramide;	translocation of		
	bronchial epithelium	phosphatidylethanolamine	drugs and		
			phospholipids		
			across the		
			membrane		
ABCC1	Alveolar type I pneumocytes;	Sphingosine-1-phosphate;	Multidrug		[55,134,179,191,200,201,202,203,204,205,208,209,210,211,212,213,214,215,216,217,218,219,220,223,224,225,232,233,234,235,236,243,244,245,248,249,250,251]
	alveolar type II pneumocytes;	leukotriene LTC_4_;	resistance;		
	endothelial cells;	phospholipid	inflammation;	Average	
	macrophages;		reducing		
	bronchial epithelium;		oxidative		
	granulocytes		stress;		
			endothelial function		
ABCG1	Macrophages;	Cholesterol;	Inflammation;	High	[69,97,121,174,184,255,257,258,259,260,261,263,264,265,266,267,268,269,270,271,272,273,274,277,278,279,280,281,282,283,284,285,286,287]
	endothelial cells;	oxysterols;	macrophages		
	alveolar type II pneumocytes;	phosphatidylcholine;	function;		
	bronchial epithelium;	sphingomyelin	apoptosis		
	airway smooth muscle cells				

## Data Availability

Not applicable.
